# Policy-Making Context Matters, But Can (and Should) It Be Operationalised? Comment on "Stakeholder Perspectives of Attributes and Features of Context Relevant to Knowledge Translation in Health Settings: A Multi-Country Analysis"

**DOI:** 10.34172/ijhpm.2022.6819

**Published:** 2022-02-14

**Authors:** Paul Cairney

**Affiliations:** Division of History, Heritage, and Politics, University of Stirling, Stirling, UK.

**Keywords:** Implementation, Policy Design, Policy Analysis, Governance, Complexity, Systems Thinking

## Abstract

Squires et al note that too many people use terms like ‘context’ imprecisely. The result (to avoid) is a catch-all term that lacks explanatory value and hinders the efforts of policy designers. Their list of 66 factors is a useful exercise to unpack what people mean when describing context. However, some problems will arise when the authors seek to move from research to practice. First, the list is too long to serve its purpose. Second, in many cases, it categorises rather than operationalises key terms. The result is the replacement of one vague term with a collection of others. Third, many categories describe what policy designers might need, rather than what they can reasonably expect to happen. In that context, wider studies of implementation and complex systems provide cautionary tales in which the outcomes of research become overwhelming rather than practical.

## Introduction

 In principle, the aim of Squires et al^1^ is laudable since they have a great general point: let’s not be too vague when we describe policy-making ‘context.’ They note that many people use ‘context’ imprecisely, often as a catch-all term for the things we suspect to be important explanations for variations across different cases. To solve this initial problem, they identify how some scholars have used this term, followed by interviews with expert practitioners in multiple countries to see what they mean by ‘context.’ The result is an impressive list, which operationalises a key idiom – forewarned is forearmed – and adds value to similar lists in the knowledge translation field. In practice, there are multiple issues worthy of further attention.

###  1. The list is too long to serve its purpose. It is overwhelming, not practical.

 While Squires and colleagues’^1^ (p. 16) initial purpose is to *foster research*, they also hope to use the results to *guide implementation* by helping ‘change agents’ to identify ‘the important features of context to consider when choosing, designing and implementing interventions.’ This aim exposes a difference between two connected objectives: to (1) *describe* or (2) *engage in* policy-making. As a *description*, a long and thorough-looking list can be interesting without being overwhelming: the reader will soon realise that one could not possibly incorporate all of these factors into policy design. Then, they might explore, for example, how policy designers simplify the list or the consequences of only taking into account a small proportion of factors. Either way, a long list is comforting to have if we do not need to use it. As an aid to *prescription*, the potential to be practical is outweighed by the likelihood that such lists become overwhelming.

 As such, the modern history of implementation studies provides a relevant cautionary tale, albeit told in more or less positive ways.^2-6^ Put most provocatively, implementation studies was ruined by an excessively long list. So called ‘first generation’ studies took a ‘top down’ approach to focus on implementation gaps with reference to a small number of key factors.^7^ These factors combined to represent a manageable research agenda (to study implementation gaps) *and* practical heuristic (to try to minimise them). The latter involved making sure that: your aims are clear and well communicated, to skilful and committed staff, while devoting sufficient resources, maintaining stakeholder support, minimising the number of actors or steps essential to the delivery chain, and hoping that external events or socioeconomic conditions do not undermine your plans. The former involved identifying a tendency – largely in case study research - for these aims to not work out in practice.

 It was followed by a ‘second generation’ of studies that examined these dynamics from the ‘bottom up.’ Such studies noted that the aim to close an ‘implementation gap’ from a top-down perspective was misguided empirically (the centre does not control implementation networks) and normatively (the ‘gap’ may be a legitimate deviation from central government aims).^8,9^

 Then came the ‘3rd generation’ of implementation scholars who sought to move beyond case studies to foster large-n studies. This task required them to turn (1) a huge shopping list of the factors that might be crucial to implementation, into (2) a shortlist that was parsimonious enough to produce a manageable research agenda. The *aim* may have been to quantify the combined impact of key factors, but the *result* was limited interest in the call for a third generation.

 Overall, what began as a simple and practical heuristic became an overwhelming list of variables. It seemed to prompt many scholars to move onto other concepts (with great potential to reinvent the wheel). Further, those who remained seemed to offer more rigour and more studies but less to say to (a diverse group of) practitioners (p. 310).^4^

 In that context, Squires and colleagues’ aim is laudable, but we should beware the unintended consequences of their attempt to solve the problem, and the possibility of creating a bigger one. This problem may be compounded by seeking new data from practitioners without first learning from previous approaches to comparable issues, including not only implementation studies but also studies of policy analysis and design.^10^

###  2. The list categorises, rather than operationalises, key factors. It cannot show what emerges when they interact.

 Implementation studies sought to operationalise key variables to help quantify the extent to which each explained implementation issues. It struggled to manage so many variables and, crucially, was not able to establish how they interacted to produce emergent outcomes in complex systems.

 In comparison, Squires and colleagues’ study *categorises*many factors, including culture, geography, governance, political climate, and leadership. Each of these concepts comes with its own literature which describes its ambiguous and multi-faceted nature. If so, much like the sorcerer’s apprentice, we may be in danger of replacing one big vague term – context – with many smaller ones.

 For example, ‘governance’ can be associated with a normative stance: the requirement for ‘good’ governance. This term is highly contested, such as when ‘new public management’ ideas based on private sector methods face some challenge from ‘new public governance’ ideas based on concepts such as collaborative governance and public value.^11-13^ Or, governance is a shorthand term to make an empirical and conceptual point, to describe the inadequacy of the word ‘government’ to describe policy-making. It is little more than a catch-all term to introduce a wide range of empirical studies in different ways.^14^

 Further, perhaps the most important category of all is ‘System complexity,’ accompanied by the quotation ‘I think a key challenge is related to the complexity or the under-estimation of the complexity of the system involved. … if you’ve done any work in a complex system, when you shift something in one place, something moves elsewhere that was unexpected’ (p. 9).^1^ It not only adds to the list of concepts that need to be unpacked to be useful, but also exposes a major division in attitudes to policy-making context. One use of ‘systems thinking’ for policy design is to seek the ability to use policy levers to produce a disproportionate impact: ‘if we engage in systems thinking effectively, we can understand systems well enough to control, manage, or influence them’ (p. 130).^10^ An alternative focus in policy studies is to describe the policy outcomes that ‘emerge’ from complex policy-making systems in the absence of central control: “we need to acknowledge these limitations properly, to accept our limitations, and avoid the mechanistic language of ‘policy levers’” (p. 130).^10^

###  3. The list identifies functional requirements alongside sources of variation.

 This distinction between types of systems thinking introduces a wider point about many of Squires and colleagues’ categories: many describe their *functional requirements* rather than actual policy-making dynamics. In the list of ‘context features,’ we find the requirement to: design a well-functioning organisational structure and networks where many actors interact, build trust through beneficial social interaction, secure organisational readiness for change, find effective local champions, secure buy-in from partners, and have sufficient capacity to deliver (including enough people well trained in implementation or ‘translation science’). There is a big difference between listing requirements and securing them in practice. As in the study of policy analysis and design, these factors may be more useful to help explain gaps between expectation and outcomes.

## Conclusion

 The authors have produced a very useful intellectual exercise, prompting scholars and respondents to be careful when using ‘context’ too loosely. This is a welcome service to the profession, which could be extended by comparing responses across different countries and political systems. However, it is not yet clear how these categories would help ‘change agents’ engage more effectively during policy implementation.

## Ethical issues

 Not applicable.

## Competing interests

 Author declares that he has no competing interests.

## Author’s contribution

 PC is the single author of the paper.

## References

[1] Squires JE, Hutchinson AM, Coughlin M (2022). Stakeholder perspectives of attributes and features of context relevant to knowledge translation in health settings: a multi-country analysis. Int J Health Policy Manag.

[2] Smith KB, Larimer CW (2009). The Public Policy Theory Primer.

[3] O’Toole LJ Jr (2000). Research on policy implementation: assessment and prospects. J Public Adm Res Theory.

[4] O’Toole LJ Jr (2004). The theory–practice issue in policy implementation research. Public Adm.

[5] Nilsen P, Ståhl C, Roback K, Cairney P (2013). Never the twain shall meet?--a comparison of implementation science and policy implementation research. Implement Sci.

[6] Cairney P. Understanding Public Policy: Theories and Issues. 2nd ed. London: Red Globe Press; 2020.

[7] Hogwood B, Gunn L. Policy Analysis for the Real World. Oxford: Oxford University Press; 1984.

[8] Hjern B (1982). Implementation research—the link gone missing. J Public Policy.

[9] Barrett SM, Fudge C. Policy and Action: Essays on the Implementation of Public Policy. London: Methuen; 1981.

[10] Cairney P. The Politics of Policy Analysis. London: Palgrave; 2021.

[11] Osborne SP. The New Public Governance?: Emerging Perspectives on the Theory and Practice of Public Governance. London: Routledge; 2010.

[12] Ansell C, Gash A (2008). Collaborative governance in theory and practice. J Public Adm Res Theory.

[13] O’Flynn J (2007). From new public management to public value: paradigmatic change and managerial implications. Aust J Public Adm.

[14] Kjaer AM. Governance. Cambridge: Polity; 2004.

